# Supplementary figures and images for: Reduced myeloid commitment and increased uptake by macrophages of stem cell–derived HPS2 neutrophils
Source: Life Sci Alliance. 2024 Jan 18;7(4):e202302263. doi: 10.26508/lsa.202302263 (PMC10796564; doi:10.26508/lsa.202302263)

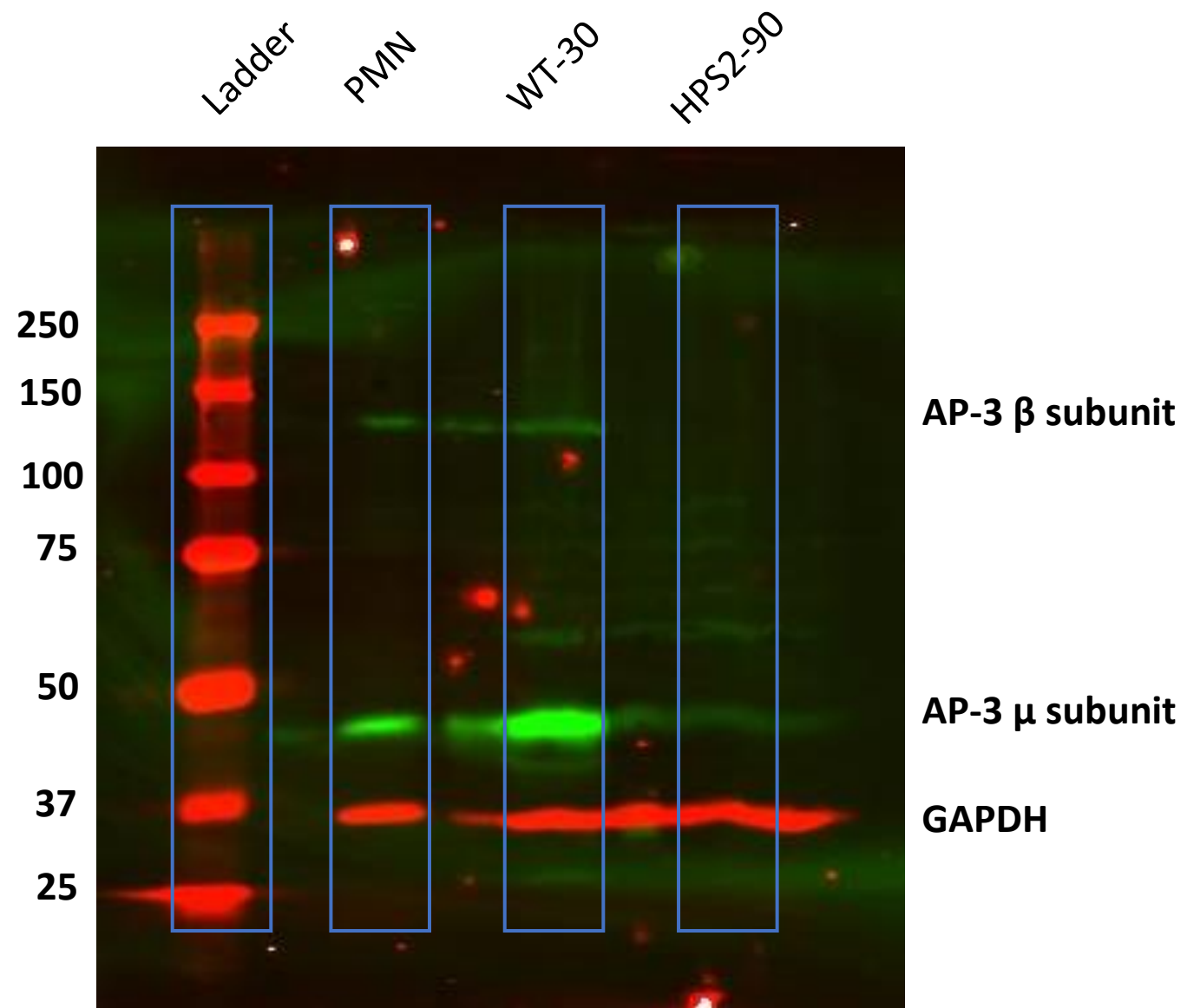

Supplement: Supplementary file 1 [file LSA-2023-02263_SdataF1.pdf]
